# Determinants of Burnout syndrome among healthcare workers in Sahloul hospital, Tunisia: A cross sectional study

**DOI:** 10.1192/j.eurpsy.2024.1235

**Published:** 2024-08-27

**Authors:** A. Fki, O. Thabet, C. Sridi, S. Ksibi

**Affiliations:** ^1^Occupational medicine, Sahloul Hospital; ^2^faculty of medicine of sousse, Sousse, Tunisia

## Abstract

**Introduction:**

Healthcare workers are at increased risk of Burnout due to the stressful demands of their job.

**Objectives:**

The aim of this study was to assess the prevalence and the related factors of burnout in healthcare workers at the Sahloul University Hospital, Tunisia

**Methods:**

Data were collected from a cross sectional study using a questionnaire exploring socio-demographic and professional data, lifestyle habits and pathological history. Burnout was assessed using the French version of the Maslash Burnout Inventory (MBI).

**Results:**

Our study included 135 healthcare workers. The average age was 41.7 ± 9.15 years. 81.5% of the sample was female. Nurses accounted for 60% of staff. More than half (51.1%) worked shifts, with night work in 32.6%. A pathological history was noted in 17.8% of healthcare workers, and a history of work-related accidents in 40.7%. The prevalence of burnout in our study population was 42.6%, with a high emotional exhaustion score in 47.4%, a high depersonalization score in 23.7% and a low personal accomplishment score in 73.3%. Burnout was significantly associated with alcoholism (p=0.016), shift work (p=0.037) and the presence of stress at work (p=0.048).

**Conclusions:**

The prevalence of burnout was high in our study population, hence the importance of setting up a burnout prevention strategy in hospitals.

**Disclosure of Interest:**

None Declared

